# An evaluation of the Kemtek 1000 sample processor

**DOI:** 10.1155/S1463924688000100

**Published:** 1988

**Authors:** M. J. Wheeler, Linzi Waiters

**Affiliations:** Department of Chemical Pathology St Thomas' Hospital London SE1 7EH UK

## Abstract

The Kemtek 1000 Sample Processor has been evaluated for precision, accuracy, speed and reliability. Precision was better than 1.0% at all volumes tested and accuracy within ±5%. A l00-tube assay could be set up within 15 min when patient specimens plus two reagents were sampled using a two probe system. Carry-over could be reduced to <0.01% by using a sufficient number of wash steps, the latter being related to the assay requirements. Evidence was found for adsorption of protein to the probe tubing but inaccuracies due to this could be reduced by introducing wash steps between samples. Problems over 12 months have been minor and quickly resolved. The authors were pleased with the way the processor performed and their staffhave confidence in leaving it to set up their assays.


Journal of Automatic Chemistry, Vol. 10, No. 1 (January–March 1988), pp. 37–42

### An evaluation of the Kemtek 1000 sample processor

**M. J. Wheeler and Linzi Walters**
*Department of Chemical Pathology, St Thomas' Hospital, London SE1 7EH*

*The Kemtek 1000 Sample Processor has been evaluated for precision, accuracy, speed and reliability. Precision was better than 1·0% at all volumes tested and accuracy within ±5%. A 100-tube assay could be set up within 15 min when patient specimens plus two reagents were sampled using a two probe system. Carry-over could be reduced to <0·01% by using a sufficient number of wash steps, the latter being related to the assay requirements. Evidence was found for adsorption of protein to the probe tubing but inaccuracies due to this could be reduced by introducing wash steps between samples. Problems over 12 months have been minor and quickly resolved. The authors were pleased with the way the processor performed and their staff have confidence in leaving it to set up their assays.*

### Introduction

The introduction of automated radioimmunoassay (RIA) systems has been slow and sporadic, mainly due to technical difficulties associated with the separation step. Most machines have also proved to be rather restricted in the type of protocol and reagent they could handle [1]. There has always been a desire for automation as routine RIA involves a great deal of manual pipetting which is tedious and repetitive. Automatic pipettes are the most common items of equipment used but their precision depends on conscientious maintenance as well as the training, temperament and expertise of the operator [2]. Diluter dispensers have relieved operator fatigue but they have not released the operator from the bench. Some laboratories have introduced pipetting stations but these have been less than satisfactory as they have not proved very flexible, and because jamming racks and blocked valves have been frequent problems.

With the advent of computer-controlled robotics a number of sample processors have been introduced to the market which can carry out the many tedious pipetting steps of RIA. These machines are able to accommodate a wide range of assay tubes, microtitre plates and reagent vessels and can potentially accept quite complicated protocols. Several hundred tubes and more than one assay can be set up in a single protocol by the larger machines.

We have had the opportunity of evaluating the Kemtek 1000 Sample Processor manufactured by the Kemble Instrument Company Ltd, Burgess Hill, Sussex, UK. The features we were interested in were its speed of operation, its ease of use, precision, accuracy and reliability.

### General description

Table 1 gives the general description of the machine with the options currently available. The computer-controlled robotic probe operates over a work platform bearing the necessary racks, beakers etc. The probe is fitted with a liquid sensing device and the dual probe option allows duplicate sampling and sequential dispensing into two assay tubes. This decreases the processing time by reducing the movements of the arm across the work area. The 'tracker ball' unit is about the size of a large paperback book and is used to set the X, Y and Z co-ordinates of the racks etc. Technical staff found it very easy and simple to use.

Several different computers are available to control the processor. Apricot microcomputers, the Amstrad PC1512HD and other IBM compatibles may be used. The type of computer chosen affects the speed of operation because delay times between operations vary.

The assay racks are a critical feature of the equipment since they must hold tubes upright with very little lateral movement. The smaller the diameter of the tube the more important this feature becomes to avoid the possibility of the probe crashing on the side of a tube. The company is able to supply purpose-built metal racks which are fairly expensive and therefore a potential user should examine

*Table 1. General description of the Kemtek 1000 Sample Processor and its accessories.*

| | |
|---|---|
| Processor | |
| Supplied with tracker ball and liquid level sensing system | |
| Net weight | 90 kg |
| Size | 1150 mm (width) × 780 mm (depth) × 500 mm (height) |
| Computer options | |
| Apricot PC 512K, dual floppy disk + 9 in monitor | |
| Apricot PC Xil0S 512K, 10 mb Winchester + 9 in monitor | |
| Apricot Xen lmb, 20 mb Winchester + 12 in monitor | |
| Amstrad PC1512HD + 12 in monitor | |
| Other IBM compatibles | |
| Options | |
| Single or two probe system | |
| Single or two pump assembly | |
| Carousel system | |
| Laser bar code reader for carousel system | |
| Magnetic stirrer | |
| Reservoir liquid level sensor | |
| Tube washer | |
| Plate washer | |
| Tube racks | |
| Dot matrix printer | |

the suitability of their own laboratory racks during the early stages of purchase.

A very comprehensive manual is provided which has simple, clear operating instructions. Details of decontamination and routine maintenance are provided and further details on trouble shooting are promised in the near future. The current programs are very flexible and can handle immunoassay protocols with a large number of steps (table 2). However, new software under development will enable the operator to program very complex protocols.

### Operating procedures

The equipment will carry out all the usual pipetting steps encountered in immunoassays using racks, microtitre plates and carousels. The X, Y and Z co-ordinates of the racks etc. have to be entered first, a procedure which takes about 10–15 minutes once the operator is familiar with the machine. Co-ordinates are stored in the computer so that this preparation needs to be carried out only once as long as the same racks are used and placed in the same position each time. This can be assured by using locating plates obtainable from the manufacturer.

Assay protocols are performed in stages by the processor. This offers great flexibility and is demonstrated by the example shown in table 2 when four beakers and three separate racks for samples, standards and quality control samples (QCs) might be employed. The manufacturer recommends that when sampling volumes of 50 µl or more, an air bubble is taken up before the sample or reagent to reduce mixing with the diluent. They recommend also that when dispensing (excluding the 'primed-through' step), the total volume of liquid plus the volume of the air bubble, plus a small volume of diluent, usually about 10 µl, should be expelled to ensure complete delivery of sample or reagents. The default volume of the air bubble and diluent is 10 µl but this can be changed by the operator. For smaller volumes, if no reagent is taken up before the sample, the manufacturer recommends taking an excess amount of sample and dispensing the required volume from it. For example, to dispense a 25 µl sample, 35 µl would be taken up and from this, 25 µl would be dispensed into the assay tube.

*Table 2. Operations carried out when setting up a LH and FSH assay in duplicate using the same samples. Four beakers contain the LH and FSH tracer and antibody and three racks held the samples, standards and QCs.*

| Stage | Tube Nos. | Reagent addition | Designation |
|---|---|---|---|
| 1 | 1–2 | 100 µl LH tracer (beaker 1) | LH totals |
| 2 | 3–4 | 100 µl LH tracer 200 µl buffer (from zero standard) | LH NSBs |
| 3 | 5–20 | 100µl LH tracer 100 µl LH standard (standard rack) | Standards |
| *4 | 21–100 | 100µl LH tracer 100 µl QCs (QC rack) and samples (sample rack) | Samples |
| 5 | 101–102 | 100 µl FSH tracer (beaker 2) | FSH totals |
| 6 | 101–104 | 100 µl FSH tracer 200 µl buffer | NSBs |
| 7 | 105–120 | 100 µl FSH tracer 100 µl FSH standard | Standards |
| 8 | 121–200 | 100 µl QCs and samples | Samples |
| 9 | 5–100 | 100 µl LH antibody (beaker 3) as primed through reagent – probe 2 | |
| 10 | 105–200 | 100 µl FSH antibody (beaker 4) as primed through reagent – probe 2 | |

* The position of QCs can be programmed when setting up the protocol.

The 'primed-through' step provides a means of rapid reagent addition. The sample line and syringe are filled with reagent fed via a separate reagent line usually from a beaker or bottle. Switching from diluent line to reagent line and vice versa is controlled by a valve system at the top of the syringe. The probe then moves from one assay tube to the next with the syringe automatically refilled via the reagent line.

### Investigations

Speed of operation – the time taken to set up an assay or series of tubes depends on a number of factors; the position of the racks, the number of reagents, the volumes to be aspirated, the number of wash steps and the total movement, up and down, as well as across the work surface. There is no program which optimizes the operating procedure of a protocol to provide the minimum time of working.

We have measured the time it takes to set up a 100-tube assay comprising two totals, two non-specific binding tubes and eight standards, three QCs and 34 patient samples in duplicate, for different protocols. The QCs were set up at the beginning and end of the samples. The time to carry out various protocols has been measured using both the Apricot Xen and the Amstrad PC1512HD, with either single or twin probe options. Usually the probe enters the assay tube to about half its depth before dispensing – this reduces any splashing of the reagent on to the side of the tube. In addition, dispensing reagent just above the tube will save the time taken for the probe to move in and out the tube and therefore reduce processing time further.

*Precision* – precision has been determined after 60 dispensings of radioactive solution at several sample volumes using a dual probe assembly. It was noticed that there appeared to be adsorption of radioactivity to the tubing and so the protein content of the radioactive solution and the diluent was varied to see if adsorption could be reduced. Precision of the 'primed-through' step was also investigated.

*Accuracy* – accuracy of sample dispensing cannot be determined using the normal procedure of dispensing as a small amount of diluent is included to ensure complete sample recovery. It is appreciated that if only the sample is dispensed or the sample plus the volume for the air bubble some loss of liquid may occur around the end of the probe and possibly through mixing with the diluent. This loss has been assumed to be very small and an estimate of accuracy has been determined by sampling different volumes of water and dispensing the sample plus the volume of the air bubble (10 μl) into preweighed tubes. After delivery the tubes were capped and re-weighed.

*Carry-over* – carry-over of a high prolactin sample to a low prolactin sample was determined. The concentration of these two samples were calculated as the mean from 10 duplicate determinations. The high sample was dispensed three times followed by three dispensings of the low sample. This was examined for each probe. The effect on carry-over when washes were included between sampling was also investigated, the usual situation in RIAs.

*Reliability* – a record has been kept of any breakdowns or problems encountered since the machine has been in routine use (now 12 months).

### Results

Table 3 shows the time taken to carry out various assay protocols with one or two washes between duplicates, and with single or dual probe assembly. There was very little difference between the two computers in the time taken to process 100 tubes; the Amstrad added no more than about 2 min to a run. Adding an extra wash step between samples increased the run time by about 2 min and using two probes decreased the time by up to 6 min. The total 'primed-through' reagent stage takes almost 8 min, but addition of reagent only takes 1·5 min, an important point if one requires fast addition of a reagent, for example, antibody, to reduce drift in an assay.

The speed of the primed-through step is related to the volume dispensed, as this governs the number of times the syringe needs to be filled during a process. Our machine

*Table 4. Time in min and s taken to dispense 100 μl or 500 μl of buffer as 'primed through reagent' into a 100-tube assay. Liquid was dispensed either at the top or half-way down the tube. Computer = Amstrad PC1512.*

| Dispensing | Volume | Prime | Dispense | Flush |
|---|---|---|---|---|
| Top | 100 μl | 4:05 | 2:04 | 1:84 |
| Half-way | 100 μl | 4:02 | 2:27 | 1:85 |
| Top | 500 μl | 4:05 | 4:26 | 1:80 |
| Halfway | 500 μl | 4:01 | 4:40 | 1:89 |

was fitted with a 750 μl syringe and therefore would dispense 7 × 100 μl aliquots but only 1 × 500 μl aliquot before refilling. If the probe has to dip inside the tube to dispense, this takes extra time. These variables have been examined in table 4. Dipping into the tubes to dispense added only 14 and 23 s to the processing time for 100 tubes whereas adding 500 μl to 100 tubes took nearly 2·5 min longer than adding 100 μl. This could be an important point in an assay susceptible to drift. Timed addition of signal reagent and reading of the signal to match the time of setting up tubes could overcome drift. However, the use of multiwell gamma counters and rapid plate readers could make such a procedure unhelpful.

Table 5 gives the precision of dispensing different volumes of radioactive solutions containing different concentrations of proteins. The recommended procedure from the manufacturer for sampling is to have a single wash step between samples to reduce carry-over. Precision incorporating this wash step has been compared with no wash step included. Precision without a wash step ranged between 0·42–1·30%CV with a median of 0·87%. With a wash step, the range was 0·16–0·68%CV with a median of 0·24%. Therefore the recommended procedure gave a precision of <0·7%.

The preset volume for the air bubble between diluent and reagent and for the amount of diluent dispensed with reagent is 10 μl. It is possible these volumes are inappropriately small for larger volumes, for example 500 μl, where greater mixing may occur, as larger

*Table 3. Times taken to process a 100-tube assay comprising totals tubes, non-specific binding tubes, eight standards, three QCs at the front and back of the assay and 34 samples in duplicate. Processing times were determined using the Apricot Xen or the Amstrad PC1512HD as the controlling microcomputer.*

| Process | | No. of washes between samples | No. of probes | Time taken (min and s) Apricot | Amstrad |
|---|---|---|---|---|---|
| Sampling only | | 2 | 1 | 12:16 | 13:30 |
| Sampling only | | 1 | 1 | 10:15 | 11:46 |
| Sampling only | | 1 | 2 | 7:45 | 9:09 |
| Sample + 1 reagent | | 1 | 1 | 14:15 | 16:15 |
| Sample + 1 reagent | | 1 | 2 | 10:30 | 11:46 |
| Sample + 2 reagents | | 1 | 1 | 18:45 | 20:38 |
| Sample + 2 reagents | | 1 | 2 | 13:08 | 14:25 |
| Primed through reagent | Prime | | | 4:20 | 4:05 |
| | Dispense | | | 1:52 | 2:04 |
| | Flush | | | 1:27 | 1:44 |
| | Total | | | 7:39 | 7:53 |

*Table 5. Precision of dispensing different volumes of iodinated testosterone in buffer with varying amounts of protein content (tracer). The liquid in the diluent line was either water or 0·1% BSA. PBS. The precision of dispensing when a single wash step was included between dispensing is also given. A dual probe assembly was used and the precision of 30 dispensings with each probe is given. Counting error has been subtracted.*

| Volume | Diluent | Tracer | Wash step | Precision (%CV) Probe 1 | Probe 2 |
|---|---|---|---|---|---|
| 25 µl | Water | 0·1% BSA.PBS* | Y | 1·39 | 0·71 |
| 25 µl | 0·1% BSA.PBS | 0·1% BSA.PBS* | Y | 0·77 | 0·48 |
| 25 µl | Water | 2·5% BSA.PBS* | Y | 1·83 | 1·15 |
| 50 µl | Water | 0·1% BSA.PBS* | N | 1·30 | 0·82 |
| 50 µl | Water | 0·1% BSA.PBS* | Y | 0·23 | 0·16 |
| 100 µl | Water | 0·1% BSA.PBS* | N | 0·55 | 0·72 |
| 100 µl | Water | 0·1% BSA.PBS* | Y | 0·21 | 0·24 |
| 100 µl | Water | 2·5% BSA.PBS* | Y | 1·01 | 0·54 |
| 500 µl | Water | 0·1% BSA.PBS* | N | 0·59 | 0·98 |
| 50 µl | 0·1% BSA.PBS | 0·1% BSA.PBS* | N | 1·03 | 0·42 |
| 50 µl | 0·1% BSA.PBS | 0·1% BSA.PBS* | Y | 0·24 | 0·36 |
| 100 µl | 0·1% BSA.PBS | 0·1% BSA.PBS* | N | 0·97 | 0·93 |
| 100 µl | 0·1% BSA.PBS | 0·1% BSA.PBS* | Y | 0·47 | 0·22 |
| 50 µl | Water | 1·0% BSA.PBS* | N | 0·89 | 0·98 |
| 50 µl | Water | 1·0% BSA.PBS* | Y | 0·63 | 0·39 |
| 100 µl | Water | 1·0% BSA.PBS* | N | 1·10 | 0·69 |
| 100 µl | Water | 1·0% BSA.PBS* | Y | 0·18 | 0·32 |
| 500 µl | Water | 1·0% BSA.PBS* | N | 0·84 | 0·84 |
| 500 µl | Water | 1·0% BSA.PBS* | Y | 0·68 | 0·65 |

* Bovine serum albumin in 0·05M phosphate-buffered saline pH 7·2.

volumes travel a greater distance along the probe tubing. We examined the effect of increasing the air bubble and diluent volumes when sampling 500 µl (table 6). Precision was not improved by increasing either of these variables.

It is conceivable that the volume delivered by each probe might be significantly different, as we found when evaluating another sample processor. The amount of radioactivity dispensed by probe 1 into 30 tubes was not significantly different (using paired 't' test) from the amount dispensed by probe 2 into a similar number of tubes. This was found to be true in 31 different experiments when volumes between 25 µl and 500 µl were dispensed.

Figure 1 shows a gradual increase in radioactivity in the first five to six tubes when sampling a radioactive solution of 0·1% bovine serum albumin in 0·05M phosphate-buffered saline, pH 7·2 (0·1% BSA.PBS) without a wash step between sampling; this occurred at all volumes. There was no related increase in weight of liquid dispensed (data not shown) and it is assumed that radioactive protein was being adsorbed to the tubing. Increasing the protein content of the reagent buffer or the diluent did not always overcome this effect, whereas including a wash step between duplicates did.

Carry-over from the high-concentration prolactin sample (120 000 mIU/l) to the following three low-concentration samples (80 mIU/l) was 4·1% in the first, 2·1% in the second and undetectable in the third tube for probe 1 and was 2·8%, 1·8% and undetectable for probe 2 when no wash was included between samples. Therefore, significant carry-over would be experienced in the two samples

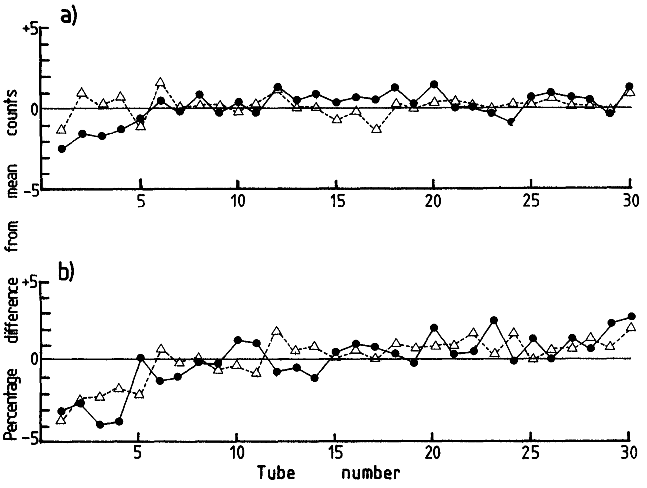

*Figure 1. Percentage difference from mean counts of 30 dispensings using differing protein concentrations in the tracer buffer and diluent. (a) tracer in 0·1% BSA.PBS, diluent = water, ●—● no wash step, △ - - △ +wash step; (b) ●—● tracer in 1·0% BSA.PBS, diluent = water, △ - - △ tracer in 0·1% BSA.PBS, diluent = 0·1% BSA.PBS, no wash step in either.*

following a very high sample. When a single wash was introduced between each dispensing, the carry-over became 0·14% for probe 1 in the first low sample tube and 0·31% for probe 2. Carry-over was not detectable in the second low tube. When two washes were introduced between each sampling no carry-over could be detected.

The weights of water collected for different sample volumes is shown in table 7 and were initially close to the chosen sample volumes. On a later occasion we found that only 450 mg water was collected for a 500 µl sample volume when using water as 'primed-through' reagent.

*Table 6. Effect of varying the volume of (a) the air bubble separating the sample and the diluent; (b) the amount of diluent dispensed with the sample. No wash step has been included between samples.*

| Volume dispensed | Volume of air bubble | Volume of diluent | Precision (CV%) Probe 1 | Probe 2 |
|---|---|---|---|---|
| 500 µl | 10 µl | 10 µl | 0·84 | 0·84 |
| 500 µl | 25 µl | 10 µl | 0·81 | 0·87 |
| 500 µl | 50 µl | 10 µl | 0·87 | 1·49 |
| 500 µl | 100 µl | 10 µl | 1·66 | 1·85 |
| 500 µl | 10 µl | 25 µl | 1·02 | 1·23 |
| 500 µl | 10 µl | 50 µl | 1·02 | 0·98 |
| 500 µl | 20 µl | 25 µl | 1·00 | 1·19 |
| 500 µl | 20 µl | 50 µl | 1·09 | 1·06 |
| 500 µl | 50 µl | 25 µl | 1·65 | 2·56 |
| 500 µl | 50 µl | 50 µl | 1·54 | 1·69 |

Further investigation showed that even for smaller volumes every time the syringe refilled, the first volume dispensed was less than expected. This proved a very repeatable error with 0·5%CV and 0·18%CV for 20 dispensings of 500 µl on two occasions. Slowing the pump speed did not alter the volume dispensed, and it was presumed that air was being pulled back along the probe line at the end of refilling. The engineer suggested that this was due to a kinked reagent line. Certainly the fault was corrected by replacing this tubing although the syringe assembly was replaced at the same time.

Reliability was good. There was an initial problem with the drive mechanism to one of the probes which was satisfactorily corrected after two weeks of investigation. There was also the temporary problem with the 'primed-through' reagent stage described above. Thirdly, the Amstrad computer had problems: after only two days' use the computer began to either cut out or had an unstable display on the monitor screen. It was suggested that this was due to a fault in the power-pack, although overheating can occur because manufacturers often insert extra boards to control their hardware. Current versions of the computer are now fitted with a fan to overcome this problem; the one evaluated was not. Kemtek's manufacturer states that they have not experienced this with other Amstrads linked to their sample processors.

### Discussion

The Kemtek 1000 Sample Processor was found to be reliable and precise. Staff are confident enough to use it as a walk-away machine. The few faults that have occurred have been quickly and satisfactorily remedied. It is important to carry out the suggested routine maintenance, which is to clean and oil the probe drive mechanism weekly and adequately flush through the tubing and syringes after use. The authors have encouraged the manufacturer to introduce default washes and suitable prompts at appropriate places to ensure adequate washing of the tubing, although to date they have not experienced any contamination in the tubing.

The possible adsorption of protein on to the tubing, and a carry-over of 2·8–4·1% when there is no wash step

*Table 7. (a) Weight of water delivered for various sample volumes. Delivery included 10µl air bubble, except for 25 µl when 35 µl was sampled and 25 µl delivered as recommended by the manufacturer.*

| Sample volume (µl) | 'N' | Weight of water (mg) ± SD |
|---|---|---|
| 25 | 20 | 24·5 ± 0·25 |
| 50 | 20 | 49·6 ± 0·75 |
| 100 | 40 | 99·8 ± 0·38 |
| 250 | 40 | 255·1 ± 2·50 |
| 500 | 20 | 500·9 ± 6·10 |
| (*b*) *Weight of water delivered as primed through reagent.* | | |
| 25 | 20 | 24·3 ± 1·30 |
| 50 | 20 | 48·4 ± 0·79 |
| 100 | 20 | 97·1 ± 0·58 |
| 500 | 20 | 496·0 ± 5·2 |

between samples, leads us to recommend a single wash step between samples. This should prove adequate for most situations and give a precision of delivery of <0·7%. However, for assays where an exceptionally large concentration range can be encountered, for example HCG, AFP and TSH assays, two washes between samples are recommended. A precision of <1·0% might be achieved by technical staff using manual pipetting but it is unlikely to be maintained for a large number of tubes over a long period of time, and assay precision may be expected to improve with the use of a sample processor.

The volume dispensed was close to the set sample volume. Accuracy experiments demonstrated that problems associated with sampling and dispensing are likely to be very repeatable and therefore not immediately noticed. The author's problem with the 'primed-through' stage resulted in poor replicates occurring in assays in a particular sequence. Therefore, should such an error occur, it will be seen as a high number of poor replicates in a particular pattern. In the authors' case, the eighth and then every seventh tube in a prolactin assay gave 10% lower counts than the other tubes.

Once a protocol is stored on the computer, operations are easy to carry out. It is therefore a good procedure to have a set of trouble-shooting protocols to test the completeness of dispensing either as a routine procedure or when problems are suspected. The amount delivered can be compared with equivalent amounts manually pipetted into tubes. This procedure is unlikely to take more than an hour.

As the sample processor is able to handle a variety of receptacles and containers it has a variety of applications. The Kemtek is likely to prove popular in routine laboratories as it is very easy to set up and use; the tracker ball is a very attractive feature. Its main disadvantage is that it is quite large, possibly larger than required for smaller district hospitals. The manufacturer has recently introduced a smaller sample processor, the Kemtek 700. As this uses the same software and simply has a smaller work area, the performance would be expected to be the same as the Kemtek 1000. At the moment the authors' are unable to recommend having an Amstrad PC1512HD microcomputer attached because it had faults. The manufacturer reports that current machines have been very reliable. The authors have experienced no problems with our Apricot Xen.

In conclusion, the Kemtek Sample Processor was found to be very easy to operate. It is both precise and reliable, and, for the routine procedures usually encountered in immunoassay, the manufacturer's preset speeds and volumes proved adequate. This saves a lot of experimentation on the part of the user to find optimum conditions. However, for very small volumes, or very viscous solutions, speed settings will probably need altering. Because the variables are infinite we have not examined the effect of changing speed settings during this study.

### Acknowledgement

This work was supported by the DHSS.

### References

1. Forrest, G. C., In: *Immunoassays for Clinical Chemistry*, Eds. Hunter, W. M. and Corrie, J. E. T. (Churchill Livingstone, Edinburgh, 1983).
2. Kilshaw, D., *Medical Laboratory Science*, **43** (1986), 378.

### FLOW ANALYSIS IV

*To be held from 17 to 20 April 1988 in Las Vegas, Nevada, USA*

Further information from Gilbert Pacey, Department of Chemistry, 112 Hughes Hall, Oxford, Ohio 45056, USA. Papers and posters scheduled for this meeting include:

Flow injection analysis today and tomorrow
*J. Ruzicka*

Aqueous flow injection analysis with FT-IR detection
*N. D. Danielson et al.*

A new approach for studying chemical interferences in flame atomic absorption spectrometry
*M. C. M. Bezerra et al.*

Determination of zinc and cadmium in small amounts of biological tissues by microwave-assisted digestion and flow injection-atomic absorption spectrometry
*J. L. Burguera and M. Burguera*

Flow injection systems with coated tubular inorganic based solid-state ion-selective electrode
*J. F. von Staden*

Flow injection analysis using a multi-ion sensor cell as detector
*P. C. Hauser*

### AUTOMATED MICROBIOLOGICAL ANALYSIS

To be held on 11 February 1988, this is a joint meeting of the Biological Methods Group and Automatic Methods Group of The Royal Society of Chemistry. The meeting will take place at the Robin Brook Centre, St. Bartholomew's Hospital, London. The programme is as follows:

Chairman's introduction
*Dr M. C. Easter (Company Microbiologist, Express Foods Group).*

AMBIS—a novel system for microbiological identification
*Professor S. Tabaqchali (St. Bartholomew's Hospital).*

Rapid enumeration of micro-organisms in biological fluids by automated fluorescence microscopy
*Dr J. Scholefield (University of Strathclyde).*

Multipoint techniques for antibiotic susceptibility, bacterial identification and radial haemolysis
*Dr P. S. Pover (Analytical Measuring Systems).*

Rapid detection and identification of bacteria in drinking-water by conductivity measurement
*G. Jones (Wessex Water Authority).*

Role of rapid microbiological techniques in the food industry
*M. R. Adams and C. F. A. Hope (Department of Microbiology, University of Surrey).*

Summing up by Dr M. C. Easter.

*Further information from the Analytical Division, Royal Society of Chemistry, Burlington House, London* W1V 0BN.

### EASTERN ANALYTICAL SYMPOSIUM

*3 to 7 October 1988 at the New York Hilton Hotel, New York City, USA*

Sponsored by the American Microchemical Society, the Society for Applied Spectroscopy, and the American Chemical Society, this is the 27th annual symposium. This year, the Eastern Analytical Symposium will be expanding its contributed presentation sessions. A 100% increase in the number of oral and poster presentations on new developments in analytical chemistry is planned. These contributions will be grouped into several sessions to complement the symposium's invited technical programme.

*Further information from Dr Stephen Scypinski, Berlex Laboratories, Inc., 110 East Hanover Avenue, Cedar Knolls, New Jersey 07927, USA.*

